# Experience in the application of laparoscopic anatomical adrenalectomy via the renal cortex surface monolayer

**DOI:** 10.12669/pjms.36.4.2102

**Published:** 2020

**Authors:** Tao Ma, Wen-zeng Yang, Zhenyu Cui, Chunli Zhao

**Affiliations:** 1 Tao Ma, Department of Urology, Affiliated Hospital of Hebei University, Baoding, Hebei, 071000, P. R. China; 2 Wen-zeng Yang, Department of Urology, Affiliated Hospital of Hebei University, Baoding, Hebei, 071000, P. R. China; 3 Zhenyu Cui, Department of Urology, Affiliated Hospital of Hebei University, Baoding, Hebei, 071000, P. R. China; 4 Chunli Zhao, Department of Urology, Affiliated Hospital of Hebei University, Baoding, Hebei, 071000, P. R. China

**Keywords:** Adrenal gland, Precise excision, Renal surface monolayer, Retroperitoneal laparoscopy

## Abstract

**Objective:**

To discuss the clinical application of laparoscopic anatomical adrenalectomy via the renal cortex surface in the operation of adrenal masses.

**Methods:**

A retrospective analysis was performed on 231 patients with adrenal masses who were received and cured in the urology department of the Affiliated Hospital of Hebei University from July 2016 to January 2019. All patients received retroperitoneal adrenalectomy by means of laparoscopic anatomical adrenalectomy via the renal cortex surface. Operation duration, bleeding volume, postoperative complications, retention time of the drainage tube were measured and analyzed, and postoperative follow-up surveys were administered.

**Results:**

All cases were successfully operated. Two cases were converted to open surgery due to the presence of large adrenal tumors, and the patients suffered no significant complications. The mean operation duration, bleeding volume and retention time of the drainage tube were 31 minutes, 20 mL and 1.2±0.6 d, respectively. In terms of the postoperative pathology of adrenal tumors, 183 cases were shown to have adenomas, 34 had pheochromocytomas, nine had schwannomas, 3 had metastases from lung cancer, and two had sarcomas. A total of 174 patients were followed up for three to 18 months. Only one case with sarcoma that relapsed within half a year of the operation.

**Conclusion:**

In the treatment of adrenal masses, laparoscopic anatomical adrenalectomy via the renal cortex surface has many advantages including the large operation space, clear view of anatomical markers, little bleeding, small trauma, few postoperative complications, simple operational procedures and short learning curves. This technique needs clinical promotion.

## INTRODUCTION

Adrenal tumors are common diseases in urology. In the case of an adrenal mass, it is necessary to determine whether the lesion is benign or malignant and whether hormone secretion is active to determine the proper therapy or follow-up visits. The European Society for Endocrinology and Ernst Guidelines offer suggestions based on the available literature. Nevertheless, there are still problems that need to be solved, such as whether to totally excise or ablate the adrenal glands, how to handle adrenal masses with unknown characteristics, and determining considerations on the duration of imaging and functional follow-up for nonsurgical patients with benign adrenal masses.^1^ At present, laparoscopic adrenalectomy is the best choice for surgical patients. Thus, precise operation and the avoidance of injury of normal tissues appear to be particularly important. Many clinicians continue studying. Good surgery planning tends to halve the work and double the results. After comparing LA and RP adrenalectomies, Lindeman^2^ noted that RP adrenalectomy is related to a short operation duration and low surgical risk for small tumors and fat indexes. Surgeons can, according to preoperative imaging, calculate and measure the distance from the skin to Gerota’s fascia, the upper boundary of the adrenal gland and kidney, the distance from the adrenal gland to the 12^th^ rib, the distance from the 12^th^ rib to the ilium, and the fat thickness and consequently decide on an operation method.^3^-^5^ Buxton^6^ treated 56 patients with HAL to assess the curative effect of this method on adrenal tumors larger than 5 cm. He noted that HAL was a safe and repeatable adrenal gland surgery method and an effective combination of a minimally invasive surgery with tactile sense. In addition, it provides a short learning curve for surgeons.^7^,^8^ In China, retroperitoneal and transperitoneal microsurgeries are widely applied.

Nevertheless, adrenal gland surgery has also evolved in various ways in recent years, such as the transabdominal, traditional retroperitoneal three-layer, prerenal fat and peritoneal monolayer, and renal dorsal psoas major approaches.^9^ The Affiliated Hospital of Hebei University innovatively adopted laparoscopic adrenalectomy via the renal cortex surface based on a summary of clinical practice experience and yielded satisfactory results. Sticking to the programmed precise operation plan, the operation method was applied in the treatment of 231 patients with adrenal gland diseases. The sections below will present the clinical effects and essentials of the operational method and the experiences in its use.

## METHODS

### Ethical approval

The study was approved by the Institutional Ethics Committee of the Affiliated Hospital of Hebei University (*IRB* 00003835, protocol 2016/17), and written informed consent was obtained from all participants.

### Inclusion criteria

(1) Surgical cases suitable for adrenal space-occupying diseases were selected according to the *Chinese Practice Guideline of Urological Diseases*; (2) laparoscopic adrenalectomy via the renal cortex surface was applied.

### Exclusion criteria

(1) Severe cardiac, cerebral, pulmonary, and hepatorenal insufficiency and severe blood coagulation disorders; (2) Retroperitoneal surgery history or infectious lesions on the affected side. A total of 231 patients with adrenal space-occupying diseases received and cured in the urinary surgery department of the Affiliated Hospital of Hebei University from July 2016 until January 2019 were included for retrospective analysis. The age of the patients was 35.6±16.1. In terms of gender, there were 129 male patients and 102 female patients. Furthermore, 143 patients presented with masses on the left adrenal gland and 88 patients on the right. The average diameter of the tumors of the patients was 2.8±2.3 cm. All patients received B-mode ultrasound and CT scanning of the adrenal cortex before surgery.

### Operation method

The celiac space was established after general anesthesia, extraperitoneal fat was removed to the fossa iliaca or removed, and the trailing edge of the lateroconal fascia/peritoneum was incised to enlarge the retroperitoneal space.

**Fig.1:**
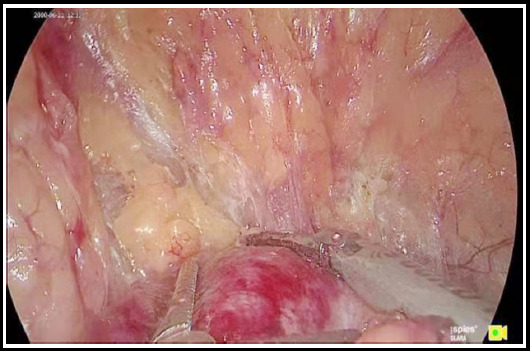
Dissociating kidney surface with ultrasound knife.

**Fig.2:**
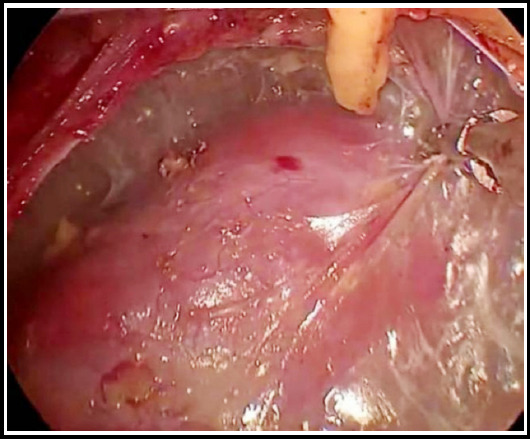
Dissociating connective tissues on the surface of kidney.

**Fig.3:**
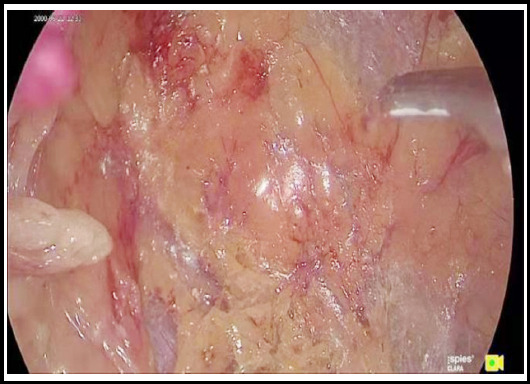
Dissociating space behind posterior peritoneum.

Immediately after the establishment and enlargement of the retroperitoneal space, perirenal fat was incised close to the peritoneal side to identify the renal parenchyma surface, which is avascular (Fig.1). The space was enlarged along this layer to dissociate the fat to the upper pole of the kidney upwards and to the inferior pole of the kidney downwards. (Fig.2) When the upper pole of the kidney was fully dissociated, the kidney was separated from the adrenal gland, and the adrenal gland area became visible when kidney was gently pressed back and downwards, while the perirenal fat, adrenal fat and adrenal gland hung above and medial to the kidney close to the peritoneum. Periadrenal fat and perirenal fat are usually of obvious elliptical slit-like structure (Fig.3). The adrenal gland can be dissociated by dissociating the periadrenal fat and adrenal gland from the perirenal fat along the slit until the peritoneum on the ventral face and psoas major on the dorsal face are reached and the two layers are enlarged and met.

**Fig.4:**
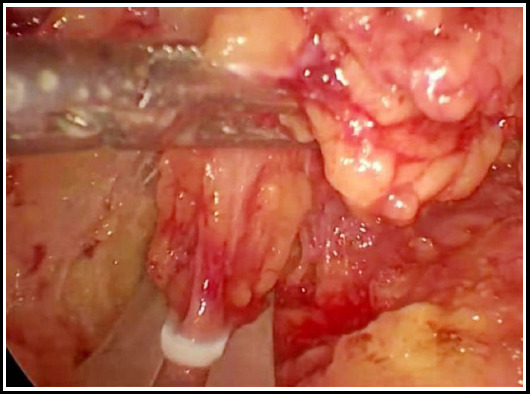
Clipping traverse adrenal gland with Hemolock clip.

The inferior angle of the adrenal gland was dissociated, the central veins were identified slightly above the inferior pole of the adrenal gland, revealed, and clipped with a Hemolock clip, and the adrenal gland and tumor were excised completely (Fig.4). For cases of a single tumor in a ventral location, surgery with partial retention of the adrenal gland is feasible, provided that the traverse adrenal gland is clipped with a Hemolock clip above the central veins. Observation and evaluation indexes were calculated for operation duration, bleeding volume, postoperative complications, retention time of the drainage tube, and postoperative follow-up.

## RESULTS

The surgeries for all 231 were successful. Two cases were converted to open operation due to the presence of a large sarcoma, and no significant postoperative complications occurred. The mean operation duration, bleeding volume and retention time of the drainage tube were 31 minutes, 20 mL and 1.2±0.6 d, respectively. In terms of the postoperative pathology of adrenal tumors, 183 cases were shown to have adenomas, 34 had pheochromocytomas, nine had schwannomas, three had metastases from lung cancer, and two had sarcomas. A total of 174 patients were followed up for three to 18 months. Except for one case with sarcoma that relapsed within half a year of the operation, no patients relapsed.

## DISCUSSIONS

At present, laparoscopic adrenal gland tumor excision is regarded as the “gold standard”. Therapy techniques for adrenal lesions via laparoscopic surgery in China are continuously updated. Urological surgeons have been trying to help clinicians acquire a shorter learning curve by imparting simpler core ideas and precise surgical skills and develop simpler and safer techniques, benefiting both clinicians and patients.^10^ Adrenal gland surgery can be conducted via the abdominal cavity or the retroperitoneal cavity. Some scholars consider the retroperitoneal cavity to be disadvantageous due to its unclear anatomic landmarks and narrow operating space, while others believed that the retroperitoneal approach was helpful for avoiding intruding into the abdominal cavity.^11^,^12^ Arezzo et al.^13^ noted that there was little evidence in the way of a comparative analysis between laparoscopic adrenalectomy via a retroperitoneal approach and transabdominal adrenalectomy. The incidence rate of tardive diseases in the case of retroperitoneal laparoscopic adrenalectomy may be lower, but the quality of evidence for this is low. Other key indexes are unknown as of yet, such as all-cause mortality, recurrence rate, economic effects, and related intraoperative and postoperative parameters.^14^-^16^ The retroperitoneal approach shows early food intake time and activity time, but long-term randomized controlled trials are necessary to acquire additional data. For the cases in this study, laparoscopic adrenalectomy via the renal cortex monolayer was conducted. With the establishment of this layer, the surgery could be performed over a large operation space, and obvious anatomic landmarks were recognizable. The operative effect was definite, so that the patients recovered quickly after the operation, could drink water properly after eight hours, and were able to walk within 24 hours.

For establishing the retroperitoneal space, the writer is accustomed to incising below the 12^th^ rib flange, dilating and pushing fat downwards from the napes with fingers, and adopting balloon dilatation. The peritoneum was kept intact when clearing the fat. Extraperitoneal fat can be placed in the fossa iliaca or removed depending on the amount of fat. The goal is to leave a large enough space and reduce disturbances. After the lateroconal fascia is opened in the internal marginal zone of the peritoneal reflection and quadratus lumborum, one key point of the surgery is to directly open the perirenal fat and conduct monolayer dissociation on a large scale along the renal parenchyma surface, with a key goal of separating the kidney from the adrenal gland. These steps and procedures are capable of yielding a clear view of the anatomy and exposing the adrenal gland area rapidly. After the exposure of the adrenal gland area, the second key goal is to recognize the boundary between periadrenal and perirenal fat and dissociate and excise the adrenal gland along this boundary. It was found that almost all patients in this study presented with this boundary as a fat slit, which is easy to recognize. Since the surgery is performed partially to dissociate along the kidney surface and to clear fat, the damage rate of the peritoneum is much lower than in the case of the traditional dissociation method via the perirenal fat and peritoneal spaces, which is advantageous for sustaining a good operation space. Regarding obese patients, the advantage of this method is that most of the thick perirenal fat hangs “in the air” along the peritoneum and is linked with the diaphragm after dissociation along the kidney surface, and satisfactory space and exposure can be obtained by clearing little or no fat. If the dissociation is made outside the perirenal fat, the fat is linked with the kidney and the adrenal gland is inside the fat; the adrenal gland can then be exposed satisfactorily by clearing much of the fat. Large adrenal tumors can be fully exposed by properly expanding the dissociation scope of the kidney to displace the kidney downwards and backwards adequately with this method. Tumors in low and deep locations and near blood vessels can also be treated because this method provides adequate exposure and a clear view of the anatomy. In the same period, single port laparoscopic adrenal surgery via the superficial layer of the kidney was conducted for five cases. Since the surgeons had little experience, the operation duration was long. Fortunately, the surgery was smooth. Agcaoglu et al.^17^ included 80 patients to compare the effect between single port laparoscopic adrenalectomy and porous laparoscopic adrenalectomy. Forty-four patients received single port laparoscopic adrenalectomy, and 36 patients received porous laparoscopic adrenalectomy. The indexes of the two groups were similar, including the average operation duration, estimated bleeding volume and tumor size. The study indicated that single port laparoscopic adrenalectomy and porous laparoscopic adrenalectomy achieved the same result with respect to postoperative short-term effects and cost effectiveness. For training young physicians, female patients of medium build with adrenal masses on the left side were selected. Three of the five young physicians successfully completed the surgery under the instruction of a senior physician, and they all considered the learning curve of this method to be short.

For patients with single tumors or adrenal lesions on both sides, we propose conducting tumor excision or partial adrenal gland excision; for multiple tumors, diffuse adrenal hyperplasia or metastatic lesions, we propose conducting total adrenal gland excision. Seyam et al.^18^ noted in their review that increasing research results suggest that the treatment effect of total tumor excision via adrenal gland surgery equaled that of radical adrenal gland excision. Partial adrenal gland excision replaces total adrenal gland excision in an increasing number of cases. Reports show that normal adrenal gland tissue can be retained regardless of whether minimally invasive surgery or an ablation technique is adopted, allowing adrenal functions to be sustained with good prognosis.

Nine of the patients in the study turned out to have adrenal schwannoma following the postoperative pathology examination. Sapalidis et al.^19^ and Sarwal et al.^20^ noted in their reports that adrenal schwannoma is a benign incidental retroperitoneal tumor that is usually diagnosed via imagological examination, particularly via CT scanning, but it is usually difficult to identify and diagnose in comparison to other malignant tumors. Adrenal schwannoma can be diagnosed definitively only by histopathological examination. Excision is considered the best therapeutic regimen. Three of the patients in this study suffered from metastatic lesions of the adrenal gland after lung cancer treatment and received this surgery for excision, which was found to have had good long-lasting results via 6-month follow-up. Drake et al.^21^ included 62 patients with metastatic tumors of the adrenal gland to conduct adrenal gland excision from 1995 to 2016, and 59 of them (95%) received laparoscopy surgery. The study endpoint was the five-year survival rate and median survival time. It was noted in this study that the procedure was safe if the excision of metastatic tumors of the adrenal gland was conducted by an experienced surgeon. The survival time of the patients receiving laparoscopic adrenalectomy to treat metastatic tumors was longer than that of patients with metastatic non-small cell lung cancer, renal cell carcinoma or melanoma who did not undergo excision of metastatic lesions.

### Limitations of the study

Exploration of single-port adrenal surgery via the superficial layer of the kidney can further reduce damage. However, the major limitation of this study is the failure to set a control group. In view of this, further observational studies are planned to compare data with other retroperitoneal and transabdominal surgical methods and provide stronger evidence for clinical promotion and application.

## CONCLUSION

Laparoscopic adrenalectomy via the renal cortex surface is a simple procedure, provides a clear view of anatomic landmarks, and has a low subsidiary-injury probability and a short learning curve. The surgical method is suitable for various adrenal lesions needing surgical treatment and is especially advantageous for patients with large or metastatic tumors or obese patient. Dissociation along periadrenal fat is helpful for reducing adrenal disturbances and avoiding fluctuations in blood pressure or heart rate during the operation. It is better to retain normal adrenal tissues as much as possible during operation, which is particularly important for patients with lesions on both sides.

### Authors’ Contributions

**TM, WZY and ZC** are responsible and accountable for the accuracy or integrity of the work.

**CZ** mainly revised the manuscript.
